# Expression of the Y-Encoded TSPY is Associated with Progression of Prostate Cancer

**DOI:** 10.3390/genes1020283

**Published:** 2010-09-14

**Authors:** Tatsuo Kido, Shingo Hatakeyama, Chikara Ohyama, Lau Yun-Fai Chris

**Affiliations:** 1 Division of Cell and Developmental Genetics, Department of Medicine, VA Medical Center, University of California, San Francisco, CA 94121, USA; E-Mail: tatsuo.kido@ncire.org; 2 Department of Urology, Hirosaki University School of Medicine, Hirosaki 036-8562, Japan;E-Mails: shingoh@cc.hirosaki-u.ac.jp (S.H.); coyama@cc.hirosaki-u.ac.jp (C.O.)

**Keywords:** prostate cancer, TSPY, EEF1A, latent cancer, clinical cancer

## Abstract

*TSPY* is a Y-encoded gene that is expressed in normal testicular germ cells and various cancer types including germ cell tumor, melanoma, hepatocellular carcinoma, and prostate cancer. Currently, the correlation between TSPY expression and oncogenic development has not been established, particularly in somatic cancers. To establish such correlation, we analyzed the expression of TSPY, in reference to its interactive oncoprotein, EEF1A, tumor biomarker, AMACR, and normal basal cell biomarker, p63, in 41 cases of clinical prostate cancers (CPCa), 17 cases of latent prostate cancers (LPCa), and 19 cases of non-cancerous prostate (control) by immunohistochemistry. Our results show that TSPY was detected more frequently (78%) in the clinical prostate cancer specimens than those of latent prostate cancer (47%) and control (50%). In the latent cancer group, the levels of TSPY expression could be correlated with increasing Gleason grades. TSPY expression was detected in seven out of nine high-grade latent cancer samples (Gleason 7 and more). The expression of the TSPY binding partner EEF1A was detectable in all prostate specimens, but the levels were higher in cancer cells in clinical and latent prostate cancer specimens than normal prostatic cells. These observations suggest that expressions of TSPY and its binding partner EEF1A are associated with the development and progression of prostate cancer.

## 1. Introduction 

*TSPY* is a Y-chromosome located and tandemly repeated gene, normally expressed in the germ cells of both adult and fetal testes [1,2,3,4]. It has been postulated to serve vital functions in male germ cell development and spermatogenesis. The TSPY gene cluster has been mapped to the critical region harboring the gonadoblastoma locus on the human Y chromosome (GBY), which predisposes the dysgenetic gonads of sex-reversed female and intersex patients to develop gonadoblastoma [5,6,7]. Indeed, TSPY is expressed abundantly in gonadoblastoma and numerous types of testicular germ cell tumors, e.g., seminoma and carcinoma *in situ*/intratubular germ cell neoplasia unclassified (CIS/ITGCNU) [4,8,9]. In addition to germ cell tumors, various somatic cancers including melanoma and hepatocellular carcinoma also express TSPY at significantly high levels, suggesting that TSPY is a cancer/testis (CT)-antigen involved in human oncogenesis [10,11]. The CT-antigens, whose expression are restricted in normal testicular germ cells and in different types of tumors, have been considered as tumor markers and promoted as target molecules for cancer vaccines [12,13]. However, only a few CT-antigens have been detected in prostate cancer [12]. We previously demonstrated that TSPY was strongly expressed in prostate cancer cells [14]. Consistently with this, it was demonstrated that TSPY expression increased in late stages of tumor progression in an independent study with the metastatic prostate cancer LNCaP cell lines [15]. Since prostate cancer involves multiple alteration of gene expression at different stages [16,17,18,19], establishing a correlation between TSPY expression and prostate cancer progression in prostate cancer specimens is crucial to understand its involvement in the initiation and progression of prostate cancer. 

TSPY is a member of TSPY/SET/NAP-1 superfamily, and shares a highly conserved NAP/SET domain with other members [20]. Members of this protein family interact with various binding partners, e.g. histones and cyclin B [21,22] via their NAP/SET domains. Recently, we identified the eukaryotic elongation factor 1A (EEF1A), a putative oncoprotein, as a novel binding partner for TSPY [23]. EEF1As are encoded by two separate genes designated as *EEF1A1* and *EEF1A2*, located on human chromosome 6q and chromosome 20q, respectively. These isoforms share >90% sequence identity at the protein level and could serve essentially the same or similar functions in protein synthesis [24]. In addition to their roles in protein translation, EEF1As are involved in various cellular functions including cell signaling and transcriptional regulation [25,26,27]. The expression levels and activities of EEF1A are correlated with cell survival and proliferation [28,29,30]. Both *EEF1A* genes have been postulated to be oncogenes involved in various types of cancer [27]. In particular, *EEF1A2* is amplified in 25% of primary ovarian tumors and is expressed abundantly in breast cancer and lung adenocarcinoma, suggesting that it could play an important role in oncogenesis in these types of cancers [31,32,33]. We previously showed that TSPY and EEF1A were co-expressed strongly in the testicular germ cell tumor cells, and demonstrated that TSPY stimulated the expression of a co-transfected reporter gene [23]. Hence, TSPY and EEF1A could synergistically exert oncogenic functions by stimulating gene expression via enhancement in both protein synthesis and gene transcription.

In the present study, we evaluated the expression of TSPY and EEF1A, with references to prostatic tumor and normal basal cell biomarkers, among non-cancer prostate (control), latent prostate cancer (LPCa) and clinical prostate cancer (CPCa) using immunohistochemistry and specific antibodies against TSPY and EEF1A. Our results suggest that expression of both TSPY and EEF1A could be correlated with oncogenic development and progression in both latent and clinical prostate cancers.

## 2. Results and Discussion

### 2.1. Characteristics of Normal Prostate, Latent and Clinical Prostate Cancer Specimens

**Figure 1:**
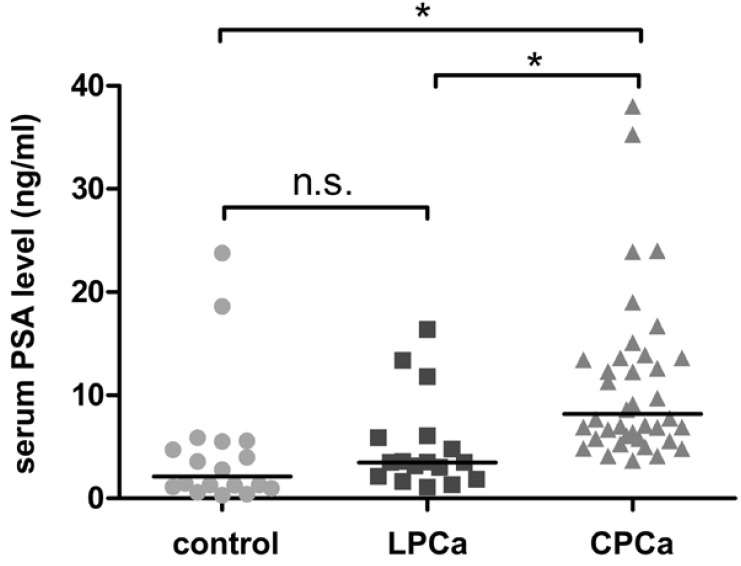
The distribution of serum PSA level in control group, latent prostate cancer group (LPCa), and clinical prostate cancer group (CPCa), respectively. The data were statistically analyzed by Mann-Whitney’s U test (see Materials and Methods). Asterisks indicate significant differences (*p* < 0.0001). ‘n.s.’ indicates no significant difference.

Table 1 shows the mean age, Gleason sum, and preoperative serum PSA level in individuals of CPCa, LPCa and control groups used in the present study. The Mann-Whitney’s U test was used to compare the age, Gleason grades and serum PSA levels among the two groups and non-cancer controls; *i.e.*, control *vs.* latent prostate cancer group, control *vs.* clinical prostate cancer group, and latent prostate cancer group *vs.* clinical prostate cancer group. There was no significant difference in the distribution of Gleason score between latent and clinical prostate cancer groups (p = 0.123). The serum PSA level in clinical cancer patients was significantly higher than the other groups (p < 0.0001; Figure 1), while there was no significant difference between the latent prostate cancer group and control (p = 0.207; Figure 1). The mean age of clinical cancer group was slightly younger than latent prostate cancer group (p = 0.032; Table 1), and there were no significant difference between latent prostate cancer group and control (p = 0.974), and between clinical prostate cancer group and control (p = 0.150).

**Table 1:** Characteristics of Clinical-and Latent-Prostate Cancer Patients and Controls.

	Controls	Latent Cancer	Clinical Cancer
	(n = 18)	(n = 17)	(n = 41)
**Mean ± SD age (years)**	69.3 ± 8.3	70.9 ± 4.2	67.6 ± 5.1^*^
**No. Gleason sum (%)**	****	****	
**GS < 7**	–	8 (47%)	17 (41%)
**GS ≥ 7**	–	9 (53%)	24 (59%)
**No. ng/ml serum PSA**	****		
**PSA < 10**	16 (89%)	14 (82%)	24 (59%)
**PSA ≥ 10**	2 (11%)	3 (18%)	17 (41%)^**^

^* ^ The age of clinical cancer patients was younger than latent cancer group (p = 0.032).

^**^     Serum PSA level in clinical cancer patients was higher than other groups (see Figure 1).

### 2.2. TSPY Expression in Human Prostate Cancer

**Figure 2:**
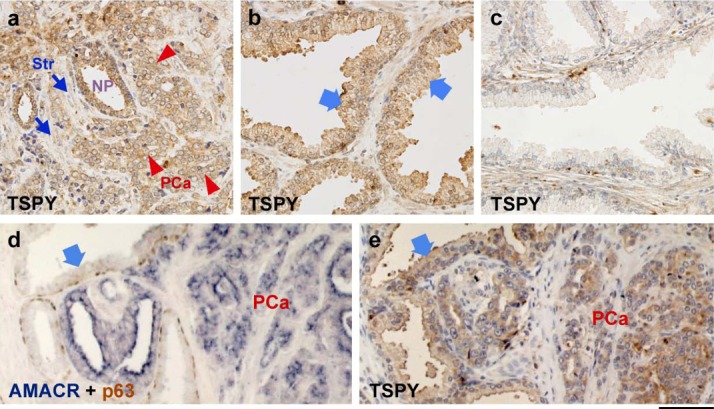
Immunohistochemical localization of TSPY and other biomarkers in the prostate tissues. **(a)** Immunostaining of TSPY (brown) in prostatic adenocarcinoma. Nuclei were counterstained by hematoxylin (purple). Note significant TSPY staining in cancer cells (red arrowheads), and faint or negative staining in the surrounding stromal cells (blue arrows). ‘NP’ indicates the TSPY-positive normal prostate gland. **(b) **Image of normal gland adjacent to cancer area in the same clinical prostate cancer section of (a). Epithelial cells in normal gland were also positively stained (arrows). **(c)** Representative image of TSPY immunostaining in the TSPY-negative gland of normal prostate. **(d)** Clinical prostate cancer section doubly immunostained for AMACR (dark blue) and p63 (brown). Arrow indicates the morphologically normal glands containing AMACR-negative epithelial cells and p63-positive basal cell layers. ‘PCa’ indicates the AMACR positive cancer area. **(e)** An adjacent section of *d* immunostained by anti-TSPY. Arrows indicate the AMACR-negative glands that were expressing TSPY. Scale bar = 75 μm.

TSPY expression was consistently detected in the majority of adenocarcinoma cells, while minimal or no expression was detected in the stromal components (Figure 2(a)) of the clinical prostate cancer group. Although in general a significantly higher frequency of TSPY expression was observed in clinical cancer samples than non-cancer control samples (Table 2), some histologically normal glands adjacent to cancer cell areas were also positive for TSPY immunostaining in the clinical cancer specimens (Figure 2(b) and Table 2). In the latent prostate cancer group, TSPY expression was positively associated with Gleason score, such that the high-grade cancer foci (Gleason grade > 7) expressed TSPY in similar frequency and levels as those in the clinical prostate cancer group (Table 3). However, our study did not show any significant association between TSPY expression and serum PSA level in any group (*p* ** > 0.22). It has been reported that the serum PSA level is not elevated in many cases of prostate cancer, even in high-grade cancer patients [34,35]. The TSPY expression may not be directly associated with the machinery for increasing the serum PSA level.

**Table 2:** The frequency of TSPY expression in prostate specimens. The ratio of TSPY-positive specimens/total are shown.

	Non cancer	Latent Cancer	Clinical Cancer
**Normal gland**	9/18 (50%)	10/17 (59%)	33/41 (80%)^*^
**Cancer cells**	nd	8/17 (47%)	32/41 (78%)^*^

^*^ The ratio is significantly higher than other groups (p < 0.05).

**Table 3:** The frequency of TSPY expression in lower Gleason grade cancer (GS < 7) and higher Gleason grades cancer (7 ≤ GS).

	Gleason score	
	GS < 7	7 ≤ GS
**Latent cancer**	1/8 (13%)^*^	7/9 (78%)^*^
**Clinical cancer**	13/17 (76%)	19/24 (79%)

^*^ The ratio is significantly different between two groups; [GS < 7] and [7 ≤ GS] (p < 0.05).

AMACR (alpha-methylacyl-CoA racemase) and p63 (a p53 homolog) have been used as indicators for cancer cells and normal glands respectively within the prostate cancer specimen [36]. AMACR is detected at high levels in the prostate cancer cells in prostate cancer specimens and has been used as a tumor biomarker. On the other hand, p63 is a basal cell marker in the prostate and is negative in cancer cells positive for AMACR (Figure 2(d)). Using these two markers, we demonstrated that TSPY could be detected in both cancer cells, associated .cancer specimens (Figure 2(e) and Table 2).

### 2.3. EEF1A Expression is Elevated in Human Prostate Cancer

**Figure 3:**
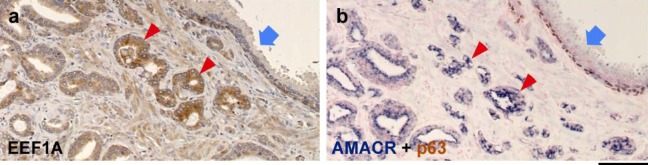
Immunohistochemical localization of EEF1A and other biomarkers in prostate tissues. **(a)** Representative image of the EEF1A immunostaining of prostate cancer specimen in which EEF1A expression was correlated with cancer development. **(b)** An adjacent section of *a* doubly immunostained for AMACR (dark blue) and p63 (brown). Nuclei were counterstained by FastRed (red). Arrows and arrowheads indicate the identical area between (a) and (b). Scale bar = 100 μm.

Eleven specimens of latent prostate cancer and 25 specimens of clinical prostate cancer were randomly selected and analyzed by immunohistochemistry with an anti-EEF1A antibody. Although there was a variation in the intensity of staining among different cell types, all examined cells were EEF1A-positive. In 30% of samples of both latent cancer group and clinical cancer group, the intensity of EEF1A-immunostaining was relatively stronger in cancer cells than normal glands (arrowheads in Figure 3(a), Figure 3(b) and Table 4). These observations are consistent with the fact that EEF1As are essential components of cell survival, despite their involvement in tumor development [27,28,29,30]. 

**Table 4:** Intensity of EEF1A in prostate cancer.

		Latent cancer	Clinical cancer
**EEF1A intensity**			
**cancer < normal**		1 (9%)	1 (4%)
**cancer = normal**		7 (64%)	16 (64%)
**cancer > normal**		3 (27%)	8 (32%)

Abbreviations: cancer, cancer cells; normal, epithelial cells in normal gland

In present study, we demonstrate that the frequency of TSPY-expression was significantly elevated in clinical prostate cancer. This finding is consistent with those in previous reports suggesting that TSPY expression increased in late stages of tumor progression [14,15]. In clinical prostate cancer group, the frequency of TSPY expression was independent of Gleason grade. On the other hand, we found that the TSPY expression was associated with Gleason grade in latent prostate cancer group (Table 3). Latent prostate cancer, by definition, is not manifested with any clinical symptoms, but is only identified in pathological analysis of specimens derived from autopsy or, in the present study, from bladder surgeries. There were no significant differences in the serum PSA level between latent prostate cancer group and control group in our study (Table 1). It has been suggested that the latent prostate cancer is likely to be a preclinical stage, and its proliferative capacity is lower than clinical prostate cancer [37,38]. TSPY expression in latent prostate cancer suggests that it is an early biomarker for initiation and/or progression of preclinical prostate cancer. Interestingly, we frequently observed expression of TSPY in morphologically “normal” glands, negative for AMACR, adjacent to cancer areas (Arrows in Figure 2(d) and 2(e)). We surmise that although both TSPY and AMACR expressions are associated with cancer development, they could be differentially regulated or activated to high levels at slightly different stages during prostatic carcinogenesis. Such expression patterns suggested that TSPY expression could be associated certain, yet-to-be defined, microenvironments involved in the initiation and progression of prostate cancer [18,19,39]. Further, since over-expression of TSPY could promote oncogenic behaviors of the host cells in both *in vitro* and *in vivo* studies [22,23,40], its expression in latent and clinical prostate cancers suggests that it may contribute to the initiation and/or progression of prostate cancer. 

Previously, we demonstrated that TSPY interacted with the putative oncoprotein EEF1A, and contributed to the cell growth properties of EEF1A [23]. Other studies also suggested that the elevated levels of EEF1A expression were associated with cell survival and cell proliferation [29,30]. EEF1A was highly expressed in various cancer types including ovarian cancer (approximately 30%), primary breast tumors (approximately 60%), and lung cancer (approximately 30%), and has been postulated as oncogenes involved in the development of various cancers [31,32,41]. The present study shows that EEF1A expression is elevated in cancer cells in 30% of prostate cancer specimens, suggesting that increase in EEF1A expression could also be associated with the progression of prostate cancer, similarly with other types of cancer. These observations support our hypothesis that TSPY and EEF1A could synergistically accelerate the tumorigenic processes in prostate cancer.

## 3. Experimental Section

### 3.1. Prostate Specimens

A total 76 prostate specimens (mean age 68.8 years, range 50–80 years), including 41 cases of clinical cancer (CPCa), 17 cases of latent cancer (LPCa) and 18 non-cancer prostates (control) were derived from archival pathological preparations of the Department of Urology, Hirosaki University, Japan. The whole prostates were fixed in 10% neutral formalin and then dehydrated with ethanol series. After the blocks of tissue were embedded in paraffin, sections were cut at a thickness of 5 μm. Sections were stained with hematoxylin and eosin (HE) for pathological evaluation and Gleason grading according to standard criteria. The preoperative serum prostate-specific antigen (PSA) level was assayed in all patients by a service clinical laboratory in Hirosaki University School of Medicine. The human study was performed under approved protocols by the respective Institutional Committees on Human Research, VA Medical Center, San Francisco and Hirosaki University, Aomori, Japan.

### 3.2. Immunohistochemistry

Immunohistochemistry was performed as described previously [23], using anti-TSPY mouse monoclonal antibody (1:3000), anti-AMACR rabbit IgG (1:50, Abcam Inc., Cambridge, MA), anti-p63 mouse IgG (1:50, Abcam), and anti-eEF1A mouse monoclonal antibody (1:100, clone CBP-KK1, Upstate, Charlottesville, VA). The immunoreactive sites were visualized by the SuperPicTure Polymer Detection kit (ZYMED/Invitrogen, Carlsbad, CA) and/or VECTASTAIN ABC-AP kit (Vector Laboratories, Burlingame, CA). The signals of TSPY-staining were amplified with Tyramide Signal Amplification Kits (Molecular Probes/Invitrogen). All immunostainings were performed by the same investigator with the same equipment setup. The Gleason scores were determined by an experienced urologic pathologist at the Hirosaki University School of Medicine, Japan. The immunostaining results were derived from consensus between the two independent experimenters/observers. Statistical analysis was performed using the non-parametric test (Mann-Whitney U-test) and Chi-square test with SPSS statistics program (Version 12; Statistical Package for Social Sciences for Windows, SPSS Inc., Chicago, IL, USA).

## 4. Conclusions

Our results suggest that TSPY expression is associated with the initiation and/or progression of tumorigenesis in both latent and clinical prostate cancers. The elevated expression of EEF1A, a binding partner of TSPY, could potentially exacerbate the cell proliferative properties of TSPY and increase the susceptibility of affected cells in oncogenic initiation and/or progression in prostate cancer.
